# Adding a back care package to the primary healthcare; a community-based cluster-randomized trial

**DOI:** 10.1016/j.bas.2023.101714

**Published:** 2023-01-20

**Authors:** Ali Ahmadzadeh Amiri, Stéphane Genevay, Amir Ahmadzadeh Amiri, Fatemeh Daneshvar, Jamshid Yazdani Charati, Mohammad Ghafouri, Navid Moghadam, Ramin Kordi

**Affiliations:** a Sports Medicine Research Center, Neuroscience institute, Tehran University of Medical Sciences, Tehran, Iran; b Division of Rheumatology, Faculty of Medicine, Geneva University Hospitals, Geneva, Switzerland; c Department of Public Health, Mazandaran University of Medical Sciences, Sari, Iran; d Health Sciences Research Center, Biostatistics Department, Addiction Institute, School of Public Health, Mazandaran University of Medical Sciences, Sari, Iran; e Spine Center of Excellence, Yas Hospital, Tehran University of Medical Sciences, Tehran, Iran

**Keywords:** Low back pain, Randomized controlled trial, Chronic pain, Incidence, Prevalence

## Abstract

**Introduction:**

The clinical course of LBP is complex and chronicity is more frequent than once thought. Moreover, insufficient evidence was found in support of any specific approach at the level of the general population.

**Research question:**

This study aimed to evaluate the effectiveness of providing a back care package through the primary healthcare system in decreasing the rate of CLBP in the community.

**Material and methods:**

Clusters were primary healthcare units with the covered population as participants. The intervention package comprised both exercise and educational content in the form of booklets. Data regarding LBP were collected at baseline, 3 and 9-month follow-ups. The LBP prevalence and the incidence of CLBP in the intervention group compared to the control group were analyzed using logistic regression through GEE.

**Results:**

Eleven clusters were randomized including 3521 enrolled subjects. At 9 months, the intervention group showed a statistically significant decrease in both the prevalence and the incidence of CLBP, compared to the control group (OR ​= ​0.44; 95% CI ​= ​0.30–0.65; P ​< ​0.001 and OR ​= ​0.48; 95% CI ​= ​0.31–0.74; P ​< ​0.001, respectively).

**Discussion and conclusion:**

The population-based intervention was effective in reducing the LBP prevalence and CLBP incidence. Our results suggest that preventing CLBP through a primary healthcare package including exercise and educational content is achievable.

## Highlights

• A community trial program consisting of both educational content and exercise trainings reduced the prevalence of Chronic LBP.
• The package helped with decreasing the incidence of Chronic LBP.
• Considerable changes in therapeutic strategies for LBP was observed.

## 1 Introduction

Low back pain (LBP) is a global health issue as it causes a substantial cause of disability (Vos et al., 2012) and work absenteeism. Considering the costs of its diagnostic and treatment procedures, LBP puts a heavy burden on the healthcare system (Vos et al., 2016, 2016v; van Tulder et al., 2000). It has been reported that similar to the developed countries, LBP is one of the leading medical problems in developing countries; in most people with a lifetime prevalence estimated between 60 and 80% (Chopra and Abdel-Nasser, 2008). Although it has been suggested that about half of LBP episodes subside within a month, the recurrence is common and, in some cases, it becomes chronic (Schaafsma et al., 2015).

Due to chronic LBP (CLBP), the patients might lose their jobs or become unable to execute their daily activities (Hoy et al., 2010). In general, most acute LBP episodes naturally subside within six weeks (Jayson, 1997). However, the clinical course of LBP is complex and chronicity is more frequent than once thought (Kongsted et al., 2015). Therefore, prevention is important both to decrease the incidence and the transition to chronicity. Nevertheless, insufficient evidence was found in support of any specific approach at the level of the general population (Chou and Huffman, 2007).

Previous studies utilizing exercise programs to prevent LBP were mostly conducted in the workplace setting and efficient results can be most expected from back-focused exercises aiming at improving back muscle and abdominal muscle strength (Schaafsma et al., 2015; Choi et al., 2010). Nevertheless, there are some shreds of evidence that exercise can reduce the intensity of LBP (Bell and Burnett, 2009). Therefore, it has been proposed that a combined program consisting of strengthening, stretching, and aerobic exercises might be effective for the prevention of LBP (Shiri et al., 2018).

On the other hand, studies demonstrated that education could play a crucial role in LBP care (Engers et al., 2008; Koes et al., 2010). However, the effect of educational interventions like back schools or advice on biomechanics which concentrate on protecting the compromised back failed to show any efficacy in lowering the incidence of LBP (Steffens et al., 2016). It has been suggested that biomedical education has limited efficacy and may even negatively affect LBP (Poiraudeau et al., 2006). On the other hand, in recent years, more attention has been paid to biopsychosocial education as a more efficient alternative. It contains information on the strength of the spine and recommendations on keeping active and how to cope with pain (Clarke et al., 2011; Louw et al., 2011). However, due to the complicated nature of human behavior, there is still no unanimity regarding the most genuine type of education.

Moreover, previous analysis (Shiri, 2017) found that a combination of exercise and education potentially could be more beneficial to prevent LBP compared with exercise alone. Consequently, we decided to implement the biopsychosocial approach plus back exercises in designing our package.

Nationwide, non-physician-primary-healthcare-providers (NPPHP) are an indispensable part of our healthcare system. They have a close relationship with their covered population and are trained to provide them with primary healthcare consults and education. Primary healthcare centers commonly distribute booklets as means to provide essential health-related materials in the community; thus, we decided to design a package for LBP prevention.

To our knowledge, there is no national educational program to prevent LBP, and also no report of a population-based program regarding LBP delivered by NPPHP. In this study, our principal goal was to assess the effectiveness of a back care package including both exercise and biopsychosocial educational content in the form of a booklet delivered by NPPHP in preventing LBP.

## 2 Material and methods

### 2.1 Design

This study is a randomized cluster field trial to investigate the effectiveness of adding a back care package for the prevention of LBP in the community. The method followed the guidelines of Consolidated Standards of Reporting Trials (CONSORT) (Campbell et al., 2012), considering the recommendations for conducting cluster randomized trials. It took place in the urban healthcare units located in the northern region of the country from December 2020 until August 2021 with follow-up evaluations at 3 months and 9 months.

### 2.2 Ethical consideration

This study was designed in accordance with the declaration of Helsinki. Informed consent was obtained from all participants and the research protocol was approved by the National Institute for Medical Research Development Ethical Committee (IR.NIMAD.REC.1398.271) and the Iranian Registry of Clinical Trials (IRCT20200707048039N1).

### 2.3 Participants

Participants were recruited from primary care practices. After fulfilling the following criteria, they were identified as eligible to enter the study: Inclusion criteria: Aged 18–75 years. Exclusion criteria: Known or suspected uncontrolled mental disorder that might prevent them from learning the contents of our educational package and precludes successful participation (e.g., schizophrenia or other psychotic conditions, bipolar disorder, major depressive disorder); known history of malignancies.

### 2.4 Sample size and randomization

After consulting with the region's healthcare headquarter, 14 primary healthcare centers (7 for each group) were invited to join the study. However, after approaching each center, 3 refused to cooperate due to the lack of time and staff. Therefore, we reassigned a cluster size of 11 to estimate the total sample size. We used the result of a recent study regarding the LBP prevalence (Noormohammadpour et al., 2016) which reported a point prevalence of 36% in the general population. We hypothesized that our intervention can reduce the prevalence to 32% at the final follow-up. With a cluster size of 11 including 5 for the control group (CG) and 6 for the intervention group (IG), an intra-cluster correlation coefficient (ICC) of 0.05, 0.8 power, and alpha of 0.05, our estimated total sample size was calculated 3310, meaning 300 subjects in each cluster. Four clusters (2 from each group) had a smaller covered population (approximately one-third of the other units), thus, about 140 subjects were assigned to be recruited from the 4 smaller clusters and 420 subjects were assigned to be recruited from the larger clusters.

### 2.5 Back care package

We designed a package consisting of 10 simple exercises (a combination of aerobics, stretching, and strengthening) that could be performed at any time at home and 7 educational points in a form of a brief illustrated story called 7 traps of low back pain. The educational booklet was developed with great care to present important tips using simple, understandable, and attractive expressions. It recommends avoiding the fear of movement, lengthy rest, not working, fear of painful days, not taking proper pain medications, thinking too much about the pain, and being nervous. Moreover, the red flags of the LBP were highlighted at the end of the booklet (a copy of the booklet alongside the translation can be found in the supplementary section).

### 2.6 Recruitment

In our country, clients routinely attend primary healthcare units for a variety of common preventive care, such as vaccination, prenatal care, and even healthcare membership extension, etc. They are mostly visited by NPPHPs and will be referred to the physician when necessary. Since our study was designed as a preventive intervention, we collaborated with NPPHPs as liaisons. The investigators thoroughly trained the NPPHPs on the aim of the study and the contents of the package in four separate sessions prior to the enrollment. Each session took approximately 60 ​min (1 session for explaining the aim of our intervention, 1 session on how to recruit the participants, 1 session on the contents of the booklets, and 1 review session). They were instructed to provide the booklets to the participants in the IG and explain the booklet point-by-point only once for 25–30 ​min. Subjects in the IG were instructed to practice the exercises regularly while following the educational tips. The subjects in the CG received their routine care and referrals if required. The potential participants were approached randomly when attending their health center to receive health-related services during our enrollment period regardless of their previous LBP status. NPPHPs were also in charge of recording the data and filling the questionnaires at baseline, 3-month, and 9-month follow-ups. The enrolled participants were asked to either return to the center for the follow-up questions or respond to the assigned NPPHP via a 20-min phone call addressing all the questions of the exact questionnaire (in case of inability to attend to the center).

### 2.7 Data collection

We prepared an online form in which all the questions were required to be answered to finish each questionnaire successfully. Therefore, it helped us to avoid missing data and made it more feasible for both NPPHP to collect data and for investigators to supervise and track the process.

### 2.8 Outcome measures

We have assessed the outcome measures using a modified National Institutes of Health (NIH) Task Force's Recommended Multidimensional Minimal Dataset for Research on Chronic Low Back Pain (Noormohammadpour et al., 2018) questionnaire recording the demographic data (age, sex, education, occupation, smoking status, marital status, and history of LBP) followed by some LBP-specific questions evaluating the primary and secondary outcomes.

### 2.9 Primary outcome

The primary outcomes were the incidence of CLBP and the point prevalence of LBP. According to the diagnostic criteria established by the NIH Task Force on Research Standards for CLBP, for the question asking “How often has LBP been a problem for you over the past 6 months?“, a response of greater than three months, or “at least half the days in the past 6 months” would define CLBP.

### 2.10 Secondary outcomes

The secondary outcomes were the intensity of LBP and the Core Outcome Measures Index (COMI) score. Patients with LBP were asked to indicate their perceived pain intensity using an 11-point visual analog scale (VAS) with 0 represents “absence of pain” and 10 represents “worst pain experienced. The Persian adaptation of COMI for back pain (Nakhostin Ansari et al., 2016) is a brief multidimensional instrument assessing the most important domains for patients with back problems (pain, function, symptom-specific well-being, quality of life, disability). The COMI has 7 questions covering 5 domains of pain intensity, function, symptom-specific well-being, general quality of life, and disability. The score (range 0–10) is calculated using the average score of the five domains (after transforming the pain score and disability question). A higher COMI score indicates a more undesirable condition. Furthermore, at each time point, we asked each participant about the management of LBP during the previous 3 months. This multiple-choice question was added to evaluate the change in the behavior of our population using the following categories: No action; Physician consultations; Imaging modalities; Medications; Surgery; Home remedies.

### 2.11 Statistical analysis

Data were analyzed according to the intention-to-treat approach. Groups were compared at baseline for demographic variables. For outcome measures, an analysis adjusted for potential confounders was performed. To assess the change of outcomes during the course of our study, logistic and linear generalized estimating equations (GEE) regression models were designed for dichotomous outcomes (i.e., prevalence) and continuous outcomes (i.e., pain intensity), respectively. The intervention effect of interest was the interaction between group and time where CG and bassline time-point were used as references. All the models were adjusted for baseline variables and possible confounders. The 95% confidence interval around the adjusted mean differences, adjusted odds ratio, and the corresponding P-value were computed. SPSS v 26 software was used for all the analyses and a significance level of P ​< ​0.05 was considered statistically significant.

## 3 Theory

We theorize that designing a comprehensive back package including both exercise training and educational content and distributing it through peripheral healthcare providers can be effective in reducing the rate of low back pain.

## 4 Results

### 4.1 Participation and loss to follow-up

Seventy-three participants of a total of 3521, including 28 in CG (13 for the first follow-up and 15 for the second follow-up) and 45 in IG (15 for the first follow-up and 30 for the second follow-up) were lost to follow-up.

### 4.2 Demographic data

The demographic data of the subjects are provided in Table 1. The percentages of female participants who were enrolled in the study were 53.66% in the CG and 56.21% in IG. The mean age of the participants was 37.7 ​± ​12.1 and 38.3 ​± ​11.6 years in CG and IG, respectively. There was a baseline imbalance regarding marital status which was not considered meaningful for potential confounders.

**Table 1:** Demographic characteristics of the subjects in each group.

VARIABLES		CG	IG	P-VALUE
**Sex (woman%)**		53.9	56.8	0.092
**Age**^**a**^		37.7 (12.2)	38.3 (11.6)	0.131
**BMI**^**a**^		26.3 (4.5)	26.4 (4.3)	0.781
**Educational status**^**b**^	**Illiterate**	62 (4.2)	92 (4.5)	0.295
	**Elementary**	228 (15.4)	345 (16.9)	
	**High school**	524 (35.5)	664 (32.5)	
	**College**	114 (7.9)	118 (5.8)	
	**Bachelor**	364 (24.6)	515 (25.2)	
	**Master**	135 (9.1)	254 (12.4)	
	**Medical doctor**	30 (2.0)	32 (1.6)	
	**PhD**	18 (1.2)	22 (1.1)	
**Occupational status**^**b**^	**Temporarily fired**	1 (0.1)	0 (0.0)	0.062
	**Disability for reasons other than LBP**	1 (0.1)	3 (0.1)	
	**Disability due to LBP**	1 (0.1)	2 (0.1)	
	**Retired**	66 (4.5)	99 (4.8)	
	**Unemployed or looking**	89 (6.0)	88 (4.3)	
	**Housewife**	453 (30.6)	694 (34.0)	
	**Employed**	736 (49.8)	1069 (52.3)	
	**Student**	131 (8.9)	88 (4.3)	
**Marital status**^**b**^	**Married**	1140 (77.1)	1734 (84.9)	<0.001
	**Single/Divorced/Widowed**	338 (22.9)	309 (15.1)	
**Smoking**^**b**^	**Yes**	121 (8.2)	158 (7.7)	0.428
	**No, quit**	23 (1.6)	20 (1.0)	
	**No, never**	1334 (90.3)	1865 (91.3)	

Data are presented as: ^a^ mean (SD) and ^b^ n (%).

### 4.3 Main outcomes

**Fig. 1:**
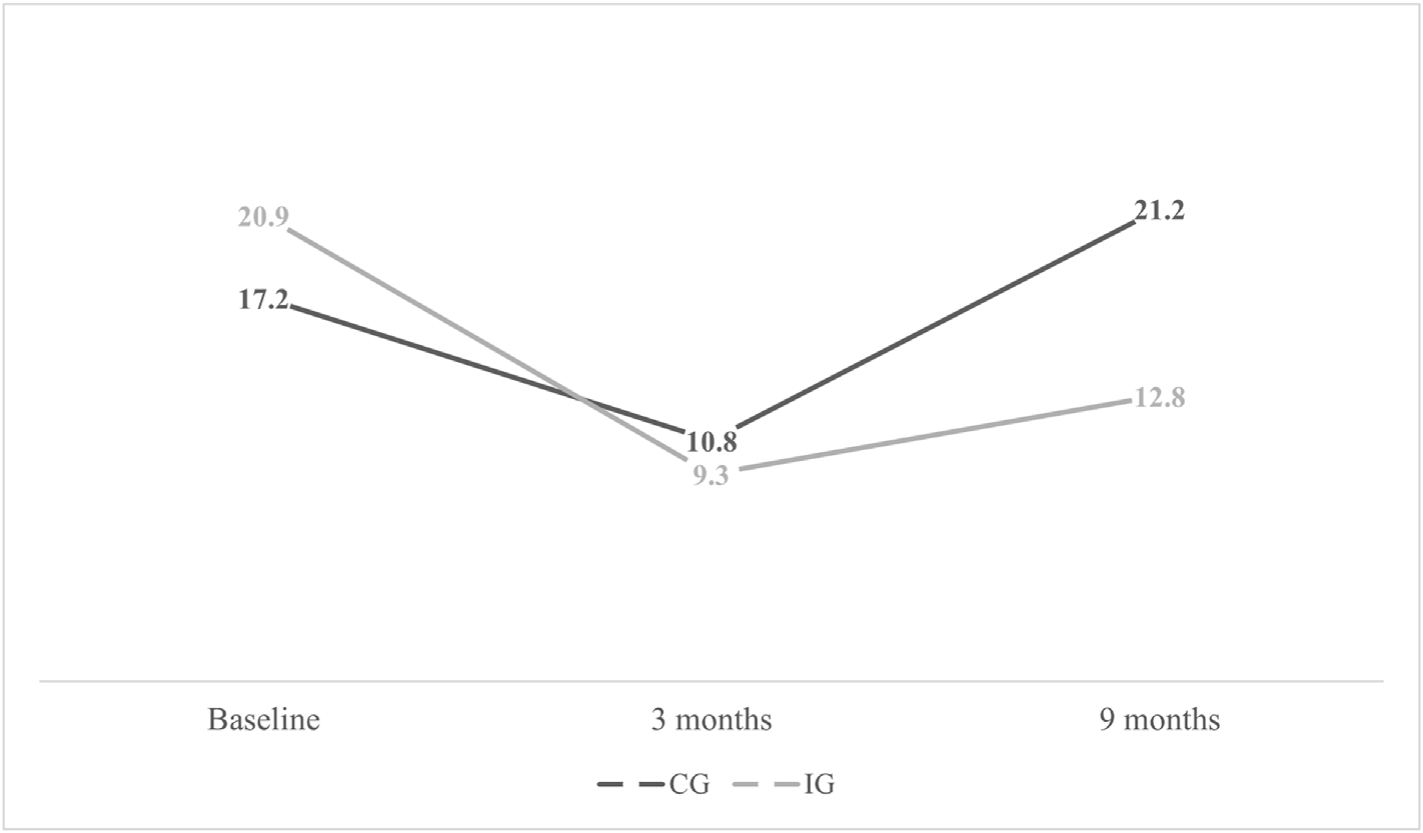
LBP point prevalence.

Fig. 1 illustrates the point prevalence of LBP during the course of our study. According to Table 2, a 57% risk reduction (OR ​= ​0.43; 95% CI ​= ​0.35–0.54) was observed at 9 months. Also, the risk of CLBP was decreased by 56% (OR ​= ​0.44; 95% CI ​= ​0.30–0.65) at the 9-month follow-up. The decrease in risk of both LBP and CLBP was statistically significant (P ​< ​0.001 and P ​< ​0.001, respectively). In addition, 89 new cases of CLBP were identified (53 (3.6%) and 36 (1.8%) in the CG and IG, respectively). Therefore, the incidence of CLBP in the IG was significantly lower compared to the CG (OR ​= ​0.48; 95% CI ​= ​0.31–0.74, P ​< ​0.001).

**Table 2:** The main outcomes of our study.

VARIABLE	TIME	CG^a^	IG^a^	Intervention effect^b^
**LBP point prevalence**^d^	**Base**	254 (17.2)	427 (20.9)	–
	**3 months**	158 (10.8)	188 (9.3)	0.66^c^ (0.51–0.86)
	**9 months**	307 (21.2)	256 (12.8)	0.43^c^ (0.35–0.54)
**CLBP point prevalence**^d^	**Base**	79 (5.3)	148 (7.2)	–
	**9 months**	74 (5.1)	64 (3.2)	0.44^c^ (0.30–0.65)
**CLBP incidence**^e^		53 (3.6)	36 (1.8)	0.48^c^ (0.31–0.74)

a Data are presented as: n (%).

b Data are presented as: OR 95% CI (odds ratio, 95% confidence interval).

c Statistically significant.

d GEE model adjusted by baseline variables was used. Reference categories were time at baseline and control group.

e Logistic regression adjusted by baseline variables was used.

### 4.4 Secondary outcomes

Table 3 provides the LBP intensity and COMI that were recorded from the symptomatic subjects at each time point. Although there was an improvement in the means of VAS and COMI at the 9-month follow-up, the improvement was not statistically significant for any of the measures.

**Table 3:** The secondary outcomes of our study.

VARIABLE	GROUP	TIME		
		BASE	3^RD^ MONTH	9^TH^ MONTH
**LBP VAS**^c^	**CG**^a^	254, 3.81 (0.12)	158, 2.58 (0.13)	307, 2.57 (0.10)
	**IG**^a^	427, 4.04 (0.10)	188, 2.69 (0.12)	256, 2.67 (0.10)
	**Intervention effect**^b^	–	0.88 (0.55–1.41)	0.88 (0.59–1.33)
**COMI**^c^	**CG**^a^	254, 3.40 (0.10)	158, 3.46 (0.12)	307, 2.88 (0.07)
	**IG**^a^	427, 3.60 (0.08)	188, 3.53 (0.11)	256, 2.86 (0.09)
	**Intervention effect**^b^	–	0.88 (0.58–1.31)	0.81 (0.60–1.10)

a Data are presented as: n, mean (SD).

b Data are presented as: OR 95% CI (odds ratio, 95% confidence interval).

c GEE model adjusted by baseline variables was used. Reference categories were time at baseline and control group.

### 4.5 Management of LBP

The treatments received by individuals suffering from LBP during the course of our study are listed in Table 4. According to the results, at baseline 287 (19.4%) subjects in CG and 457 (22.4%) subjects in IG reported suffering from LBP in the last 3 months. In addition, at the first follow-up, 256 (17.5%) subjects in CG and 278 (13.7%) in IG reported having LBP during the 3 months between baseline and first follow-up. At the last follow-up, 501 (34.6%) in CG and 534 (26.7%) in IG reported back pain during the previous 3 months. Considering the three months intervals before each time point, for most of the studied variables, we observed significant changes in therapeutic strategies for LBP between the two groups with a decrease in medical consultations, use of imaging, and medication. There was no significant change in the proportion of participants reporting having done nothing for their back pain. Concerning surgery, also some differences are reported, numbers are too low to be relevant.

**Table 4:** The therapeutic measures against LBP.

MEASURES	GROUP	TIME		
		Base	3^RD^ MONTH	9^TH^ MONTH
**No action**^d^	**CG**^a^	51 (17.8)	88 (34.4)	144 (28.7)
	**IG**^a^	70 (15.3)	104 (37.4)	138 (25.8)
	**Intervention effect**^b^	–	1.43 (0.86–2.37)	1.07 (0.68–1.68)
**Physician consultation**^d^	**CG**^a^	115 (40.1)	30 (11.7)	129 (25.7)
	**IG**^a^	231 (50.5)	38 (13.7)	98 (18.4)
	**Intervention effect**^b^	–	0.74 (0.41–1.36)	0.45^c^ (0.30–0.68)
**Imaging**^d^	**CG**^a^	76 (26.5)	8 (3.1)	74 (14.8)
	**IG**^a^	142 (31.1)	23 (8.3)	33 (6.3)
	**Intervention effect**^b^	–	1.69 (0.74–3.83)	0.32^c^ (0.19–0.54)
**Medication**^d^	**CG**^a^	144 (50.2)	83 (32.4)	205 (40.9)
	**IG**^a^	216 (47.3)	81 (29.1)	143 (26.8)
	**Intervention effect**^b^	–	0.94 (0.60–1.49)	0.61^c^ (0.41–0.90)
**Surgery**^d^	**CG**^a^	5 (1.7)	1 (0.4)	0 (0.0)
	**IG**^a^	8 (1.8)	1 (0.4)	9 (1.7)
	**Intervention effect**^b^	–	–	–
**Home remedies**^d^	**CG**^a^	98 (34.1)	127 (49.6)	184 (36.7)
	**IG**^a^	112 (24.5)	120 (43.2)	279 (52.2)
	**Intervention effect**^b^	–	1.22 (0.81–1.86)	2.38 c (1.67–3.41)

a Data are presented as: n (%).

b Data are presented as: OR 95% CI (odds ratio, 95% confidence interval).

c Statistically significant.

d GEE model adjusted by baseline variables was used. Reference categories were time at baseline and control group.

## 5 Discussion

### 5.1 General findings

The purpose of this study was to determine the effect of a prevention program provided in the primary health care setting by delivering a back pain package on lowering the risk of LBP and CLBP. The results showed that educating patients through NPPHPs providing the designed booklet that included both exercise and educational content not only reduced the point prevalence of LBP and CLBP but also decreased the incidence of CLBP at 9 months.

### 5.2 Primary outcomes

The observed reduction of the risk of LBP and CLBP supports our hypothesis that a community-based intervention through NPPHPs could be beneficial for the general population. Although studies on prevention programs implementing exercises alone have not always been successful (Schaafsma et al., 2015), others have found similar results as ours (Sihawong et al., 2014). It is also possible that the contents provided by the education part had a significant amplifying effect. Our most important finding was the significant decline in the incidence of CLBP which decreased the risk by 52% in our population after 9 months. Although there are still scarce interventions directly measuring the incidence of CLBP, a 57% significant decrease in the point prevalence of LBP in our study is in line with the systematic review by Shiri et al. 12 who reported that exercise combined with education reduced the risk of LBP by 27% (confidence interval 9–41%). The difference in the magnitude of the effect could be due to different methodological approaches such as the targeted population, the details of the exercises, and the educational contents.

Biomedical education has been shown to increase attention to pain and have a deteriorating impact on pain sensation (Main et al., 2010). On the contrary, it has been indicated that neuroscience education, which is based on biopsychosocial education, may be effective in treating chronic musculoskeletal pain and LBP. Previous studies by Moseley et al. (Moseley, 2004; Moseley et al., 2004) suggest that biopsychosocial education generates more preferable outcomes to biomedical education. Therefore, considering the controversial debates on the effects of biopsychosocial compared with biomechanical education, we assume designing our package based on the biopsychosocial approach plays an important role in the findings of our study.

Suni et al. (2013) conducted a trial to investigate the effectiveness of an exercise and counseling program for reducing the incidence of LBP in young healthy Finnish conscripts at the beginning of their compulsory military service and found a significant 58% decrease in the incidence of LBP. Also, in a study by Tonosu et al. (2016) it was revealed that an exercise program followed by an educational manual was effective in preventing LBP in Japanese healthcare workers.

### 5.3 Secondary outcomes

Although it was revealed that IG had suffered less pain intensity at the follow-ups compared to CG, the results did not show any statistically significant improvement. We also observed a decreasing trend in the COMI scores in both groups and the difference between the two groups was not statistically significant. We are aware that both VAS and COMI measures were below the pathological thresholds. Therefore, it was predictable that our intervention might not be effective on pain intensity and disability since in our country most patients with considerable back pain would visit hospitals or tertiary centers instead of primary healthcare units. Our recruitment centers are mostly the destination for patients with mild to moderate back pain and disability. Although most interventions on LBP measuring pain intensity and disability are focused on the treatment of LBP (Bell and Burnett, 2009), our findings follow the results of a previous systematic review conducted by de Campos et al. (de Campos et al., 2021) which concludes for a shred of moderate-quality evidence that a program combining exercise and education is not effective in reducing future LBP intensity and associated disability in long-term. Similar to our study, Kamioka et al. (2011) conducted a trial implementing exercise and education on female Japanese caregivers in nursing homes and reported that no significant differences were seen for LBP intensity. Warming et al. (2008) also revealed that an educational program combined with exercise was not effective in reducing the LBP intensity and related disability in Danish nurses. Again, these conflicting results may be due to the differences in the methodology and design. We designed a pragmatic cluster trial encouraging the general population to exercise regardless of their LBP status, while other trials were concentrated on supervised cognitive or physical therapies for LBP patients. Also, we utilized COMI as a thorough indicator of pain, disability, and quality of life, whereas most studies used other isolated disability measures.

In this study, we measured both point prevalence and a 3-month interval prevalence before each time point. The former was used as a part of our primary outcome (Table 2). However, the latter was used to have a more accurate understanding of the changes in the therapeutic strategies during that time. Our package was designed to provide some tips on how to both avoid and relieve LBP. Therefore, we were curious whether we could help subjects take proper measures to deal with the pain or not. Our study showed that our back care package was able to modify the way the participants dealt when experiencing an episode of back pain. In the intervention group, we observed a significant reduction in the number of individuals visiting the physician, having imaging, and taking pain medicine. All these interventions are important components of costs related to back pain. These findings suggest that an educational plus exercise package could possibly decrease the important burden LBP management is putting on the healthcare systems. These promising results are certainly encouraging further investigations focusing on the cost-effectiveness of our back care package.

### 5.4 Strengths and limitations

One of the strengths of this study is the cluster randomized controlled trial design, which is considered the best methodological design for the evaluation of population-based health interventions. Furthermore, the innovation in providing booklets through NPPHPs was another point of strength as booklets were made to be easily accessible providing back-focused exercises alongside educational content based on the best current scientific pieces of evidence on the biopsychosocial model. The proximity of the NPPHPs to the population is certainly an important point to support the dissemination and implementation of the recommendations. Also, our design made it possible for us to include participants regardless of their previous LBP condition, occupational status, or any other limiting factor so that our study could better represent the community. The online data collection process was one of the strengths of our work as it helped us prevent data loss in each questionnaire.

On the other hand, are findings showed that there was a baseline difference in the prevalence of LBP between the two groups, which could be justified by the limitations in the recruitment process through designated liaisons. However, at the final follow-up, we witnessed a significant decrease in IG compared to a slight increase in CG after 9 months which only reflects more on the impact of our intervention. Also, due to the population-based nature of our design, it is impossible to further analyze the intermediate mechanisms allowing for such a positive effect nor can we determine the respective effects of exercises and education on the global effect. In other words, we could not specify which component of our intervention was the most effective. Since lifestyle modifications are difficult to implement and measure, we could not identify the adherence of the IG to both parts of our booklet, separately. It was only possible to measure how much did they adhere to the exercise component. The impact of the educational component was objectively immeasurable. It largely depends on each participant's ability to absorb the information and it requires different study designs and measurements. Further studies are needed to measure the adherence of the participants to the recommendations more accurately. In addition, we had no information about additional services that participants could have received outside the study. Although randomization and the number of participants should decrease this kind of bias, we cannot rule out the influence of any other kind of therapy or treatment. Finally, even though the clusters were independent and each unit was located in a different region of the city, contamination cannot be ruled out. However, this could only strengthen our findings.

## 6 Conclusion

In conclusion, our results suggest that a population-based intervention was effective in reducing the LBP prevalence and CLBP incidence. Our results suggest that preventing CLBP through a primary healthcare package including exercise and educational content can be achievable in the community and might decrease the high level of burden LBP is putting on the health systems.

## Funding

This study was funded by 10.13039/501100012155National Institute for Medical Research Development (NIMAD).

## Ethical code

IR.NIMAD.REC.1398.271.

## RCT registration number

IRCT20200707048039N1.

## Declaration of competing interest

The authors declare that they have no known competing financial interests or personal relationships that could have appeared to influence the work reported in this paper.

## Appendix A Supplementary data

Multimedia component 3 Multimedia component 3

figs1 figs1

figs2 figs2
