# Supplementary material for: Adding a back care package to the primary healthcare; a community-based cluster-randomized trial
Source: Brain Spine. 2023 Jan 20;3:101714. doi: 10.1016/j.bas.2023.101714 (PMC10293304; doi:10.1016/j.bas.2023.101714)
Supplement: Multimedia component 1 [file mmc1.docx]

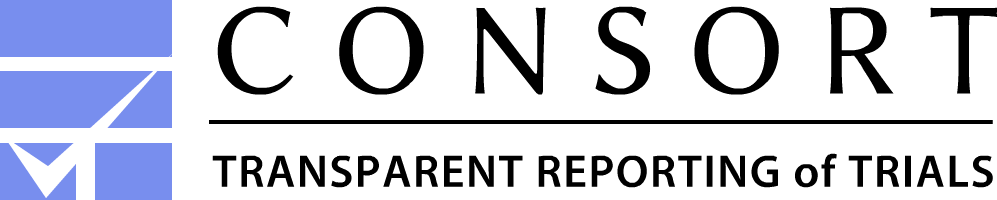


**CONSORT extension for cluster trials flow diagram**

## Enrollment

Eligible primary healthcare centers invited to participate (n=14)

Randomized centers (n=11)

Primary healthcare centers excluded, no time (n=3)

Allocated to intervention (n=6)

- Received: Average cluster size 341, range 170-641, total 2043

- Did not receive: (n=0)

Allocated to control (n=5)

- Received: (n=5) Average cluster size 296, range 129-447, total 1478

- Did not receive: (n=0)

## Allocation

## Follow-Up

15 participants lost to 1st follow-up, no response

Average cluster size 338, range 178-648, total 2028

13 participants lost to 1st follow-up, no response

Average cluster size 293, range 129-441, total 1465

## Follow-Up

30 participants lost to 2nd follow-up, no response

Average cluster size 333, range 172-634, total 1998

15 participants lost to 2nd follow-up, no response

Average cluster size 290, range 124-439, total 1450

## Analysis

Analyzed

Average cluster size 333, range 172-634, total 1998

Analyzed

Average cluster size 290, range 124-439, total 1450
