# Supplementary material for: Adding a back care package to the primary healthcare; a community-based cluster-randomized trial
Source: Brain Spine. 2023 Jan 20;3:101714. doi: 10.1016/j.bas.2023.101714 (PMC10293304; doi:10.1016/j.bas.2023.101714)
Supplement: Multimedia component 2 [file mmc2.docx]

**7 Traps of low back pain (LBP)**

**Introduction:**

Mr. Bijan is an office employee and her wife Mrs. Mojdeh is a housewife. It has been more than 2 weeks since they are suffering from LBP. Therefore, they went to their doctor’s office for help. After examination, the doctor told them that their problem is a simple mechanical LBP and they should not worry. However, they must avoid **7 traps** to relieve the pain.

**Trap #1**

- What kind of traps doctor? I am afraid of doing something to make it worse! I didn’t even move for as long as possible!
- Don’t be afraid Mr. Bijan! Your spine is like a door hinge. The more still you keep it, the rustier it gets! So, the first trap is the **fear of moving.**

**Trap #2**

- But I heard resting is good for your back!
- Yes Mrs. Mojdeh, but not too much rest! - Your back doesn’t need long rests. You must return to your normal daily activities steadily after a couple of days of resting. It mustn’t take you more than a week to get back to your daily routines. So, **resting too much** is the second trap.

**Trap #3**

- So you’re saying I made the mistake to lay in bed for 2 whole weeks and didn’t go to work?
- Indeed Mr. Bijan! You have to inform your boss so that they can manage easier tasks for you in the first couple of days but you should not be absent from work. You to Mrs. Mojdeh. You should have started doing the chores slowly. It both helps your body and mind. So, the third trap is **not working.**

**Trap #4**

- You see doctor, some days I have so much pain that I can’t perform at all!
- Well, Mrs. Mojdeh you have to know that it's only natural that you experience some more painful days in the course of your LBP. But you have to be aware that in most cases LBP is just like a common cold! Plenty of people suffer from it and yet perform their daily tasks. So, instead of doing nothing, you have to learn to adapt and work with a lower intensity until it’s resolved. So, the fourth trap is **fearing the painful days.**

**Trap #5**

- How should we deal with this pain then? These medications can’t be good! They only numb the pain. I heard it makes you not notice the pain and hurt your back even more!
- Don’t make a mistake here Mr. Bijan! Pain pills are the first choice in your treatment plan. They not only reduce your pain but also reduce the inflammation in your back. If you take them properly following your doctor’s prescription, they even won’t have any serious side effects. You can also use other remedies such as hot packs or anti-pain ointments. So, the fifth trap is **not taking pain pills.**

**Trap #6**

- But you see doc, what if I have a serious problem in my back? I don’t think it will get better any time soon.
- Mr. Bijan, you must acknowledge that sometimes we get too sensitive about our pains. In other words, even when we don’t have a serious problem, we feel the pain more often and more intensely than we should. That forces our body to take longer to heal. So, the sixth trap is **thinking too much about the pain.**

**Trap #7**

- But I’m so nervous about it! One of our neighbors had the same kind of pain and she had to undergo surgery. I don’t want that.
- Mrs. Mojdeh, the more nervous you get, the more sensitive you would be to the pain. I am sure you can find examples of people who are calm and whose pain vanished more quickly. If you worry all the time other problems can add up to your LBP.
- The doctor is right Mojdeh! We have a colleague at the office who despite all the struggles is always relaxed! We never saw him sick or in pain!
- Exactly Mr. Bijan! Then the last trap is **being nervous.**

**Red flags of LBP**

Remember to consult with your doctor if your pain starts after a trauma or an accident, you have numbness in your legs with your pain, you have a fever, weakness, or weight loss accompanying your pain, or the pain wakes you up from sleep.
