# Supplementary material for: Adding a back care package to the primary healthcare; a community-based cluster-randomized trial
Source: Brain Spine. 2023 Jan 20;3:101714. doi: 10.1016/j.bas.2023.101714 (PMC10293304; doi:10.1016/j.bas.2023.101714)
Supplement: Multimedia component 3 [file mmc3.docx]

**10 exercises for low back pain (LBP)**

**Introduction**

LBP is a common problem causing many discomforts and limiting activity. You can strengthen your muscles by performing these 10 exercises and prevent or relieve your LBP. You must perform them 5 days a week in 3 to 5 repetitions a day. Start slowly and increase the intensity gradually. It might cause some temporary pains at first which is natural, but if it continued to bother you or worsened your pain avoid that particular exercise and call your doctor.

1. Walk or run slowly in your home, office, or yard for 3 to 10 minutes.
2. Lay on your stomach, put your hands on the ground next to your head, and while your hip is touching the ground move your upper body upwards slowly. Hold it for 3 to 5 seconds and go back down.
3. Lay on your stomach, put your arms under your chin, fold your knees and contract your gluteal muscles for 3 to 5 seconds. Do this for each knee separately.
4. Sit on your knees, put your hands down on the ground, bow your head down, and make an arch with your back. Hold this position for 3 to 5 seconds then move your head upwards and make a reverse arch with your back and hold it for 3 to 5 seconds too.
5. Lay on your back, tuck one of your knees with your hand and hold it close to your stomach for 3 to 5 seconds. You have to do this exercise for each leg separately.
6. Sit on your knees, put your hands down on the ground and slowly move your hip forward, hold it for 3 to 5 seconds.
7. Lay on your back. Put your hands beside your body and tuck your knees. Move your knees left and right until they reach the ground on each side. Hold them down for 3 to 5 seconds.
8. Sit on your knees, and put your hands down on the ground. Move up your left hand and right leg simultaneously aligning your body, hold for 3 to 5 seconds and do the same for the opposite arm and leg.
9. Lay on your back. Put your hands beside your body and tuck your knees. Lift your hips upwards slowly, hold for 3 to 5 seconds and bring it down again.
10. Stand up, slowly tuck your knees and sit until you touch the ground, hold for 3 to 5 seconds and stand up again.
