# Supplementary figures and images for: Adding a back care package to the primary healthcare; a community-based cluster-randomized trial
Source: Brain Spine. 2023 Jan 20;3:101714. doi: 10.1016/j.bas.2023.101714 (PMC10293304; doi:10.1016/j.bas.2023.101714)

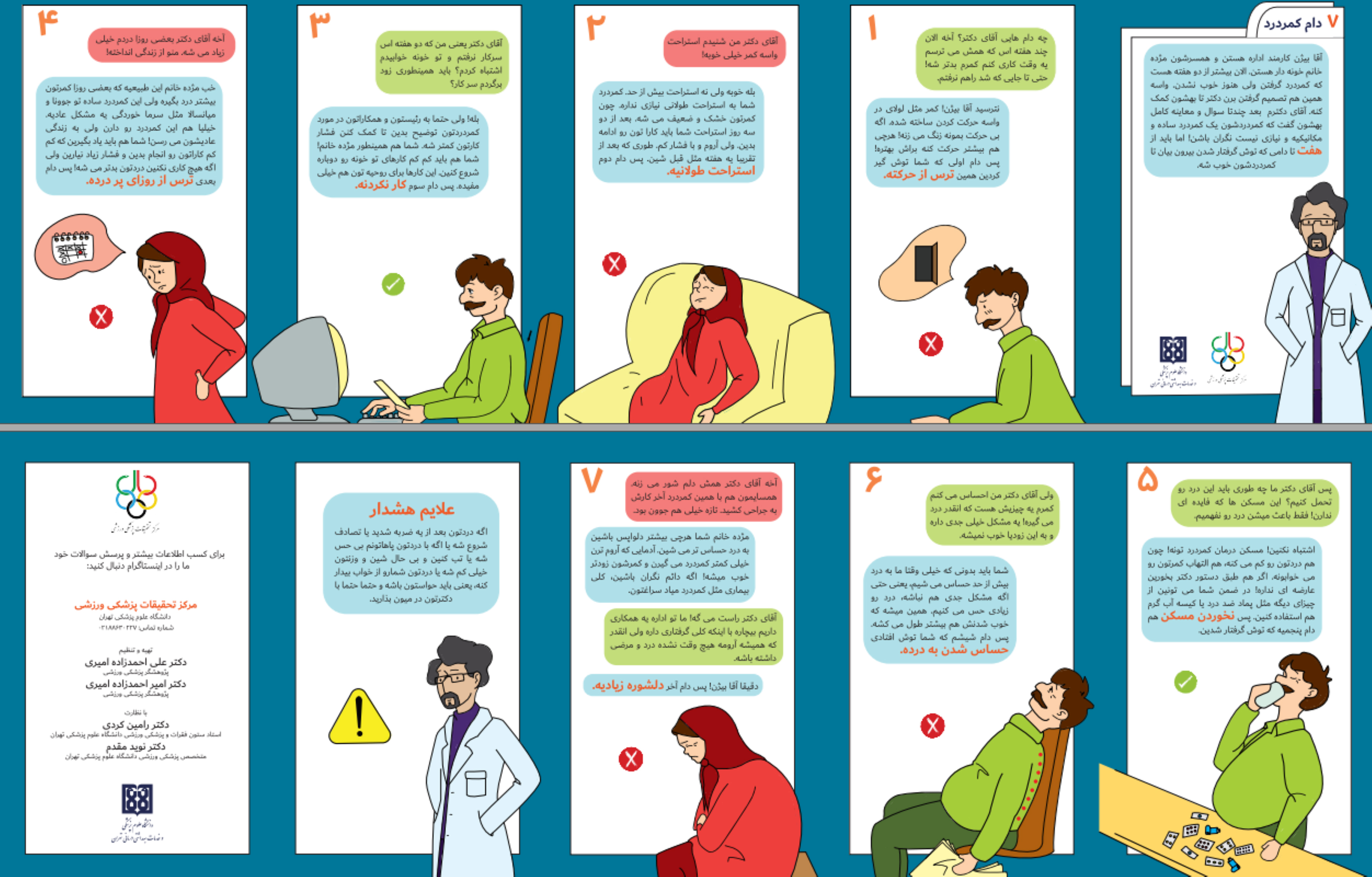

Supplement: figs1 [file figs1.jpg]

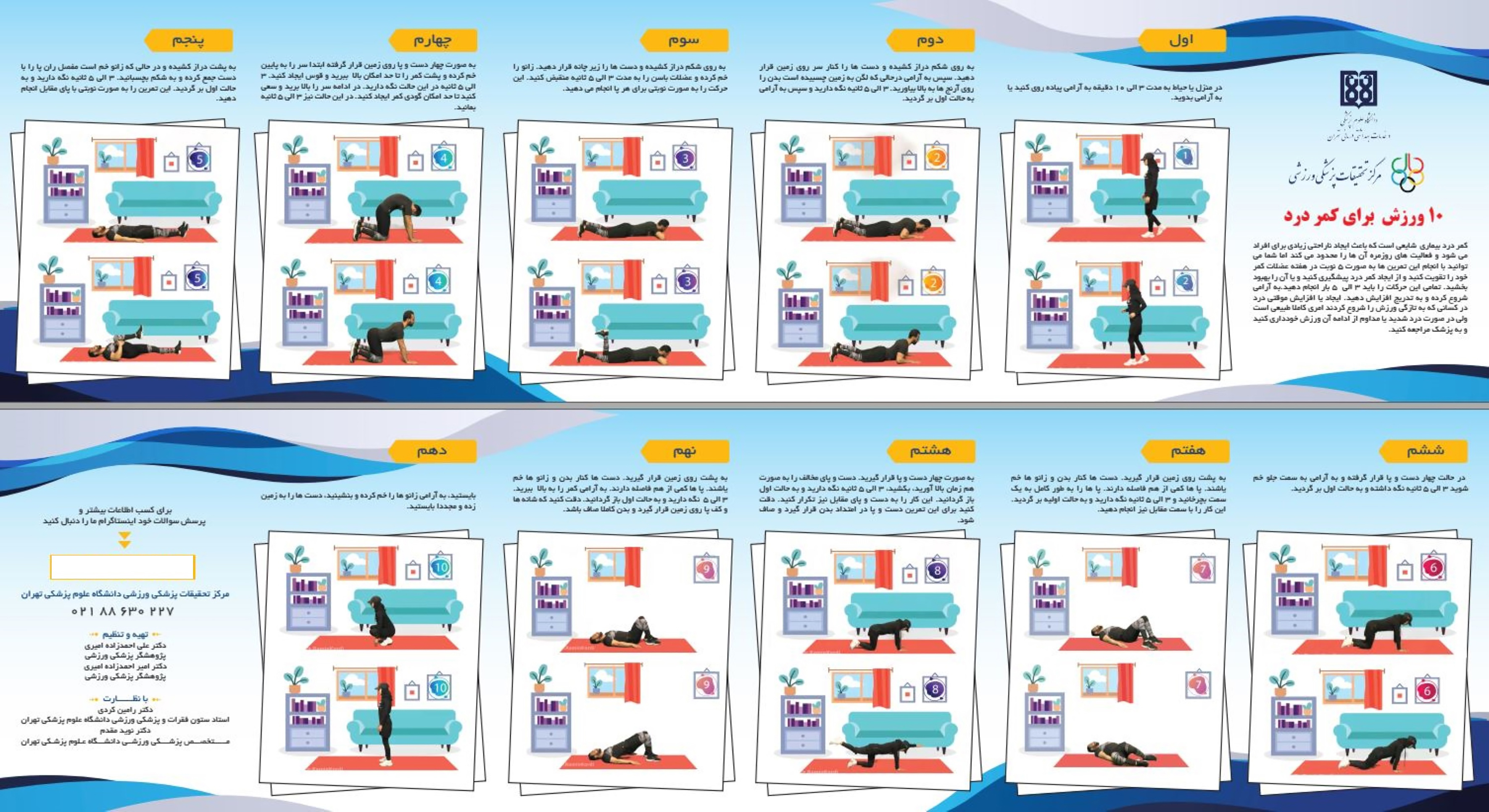

Supplement: figs2 [file figs2.jpg]
